# fLPS: Fast discovery of compositional biases for the protein universe

**DOI:** 10.1186/s12859-017-1906-3

**Published:** 2017-11-13

**Authors:** Paul M. Harrison

**Affiliations:** Department of Biology, McGill University, Montreal, QC Canada

**Keywords:** Composition, Bias, Low-complexity, Annotation, Protein, Intrinsic disorder, Prion

## Abstract

**Background:**

Proteins often contain regions that are compositionally biased (CB), i.e., they are made from a small subset of amino-acid residue types. These CB regions can be functionally important, e.g., the prion-forming and prion-like regions that are rich in asparagine and glutamine residues.

**Results:**

Here I report a new program fLPS that can rapidly annotate CB regions. It discovers both single-residue and multiple-residue biases. It works through a process of probability minimization. First, contigs are constructed for each amino-acid type out of sequence windows with a low degree of bias; second, these contigs are searched exhaustively for low-probability subsequences (LPSs); third, such LPSs are iteratively assessed for merger into possible multiple-residue biases. At each of these stages, efficiency measures are taken to avoid or delay probability calculations unless/until they are necessary. On a current desktop workstation, the fLPS algorithm can annotate the biased regions of the yeast proteome (>5700 sequences) in <1 s, and of the whole current TrEMBL database (>65 million sequences) in as little as ~1 h, which is >2 times faster than the commonly used program SEG, using default parameters. fLPS discovers both shorter CB regions (of the sort that are often termed ‘low-complexity sequence’), and milder biases that may only be detectable over long tracts of sequence.

**Conclusions:**

fLPS can readily handle very large protein data sets, such as might come from metagenomics projects. It is useful in searching for proteins with similar CB regions, and for making functional inferences about CB regions for a protein of interest. The fLPS package is available from: http://biology.mcgill.ca/faculty/harrison/flps.html, or https://github.com/pmharrison/flps, or is a supplement to this article.

**Electronic supplementary material:**

The online version of this article (10.1186/s12859-017-1906-3) contains supplementary material, which is available to authorized users.

## Background

Proteins are (usually) made from an alphabet of twenty amino acids. However, these are not represented democratically in every sequence. Some short protein sequence tracts may only use a small subset of the possible amino-acid residue types and thus have a compositional bias (CB), e.g., the tract QHQQQGQHHQHHHQQQQHH has a multiple-residue bias for Q (glutamine) and H (histidine). Such tracts are often called ‘low-complexity sequence’. Also, a protein may be compositionally biased for a small number of residue types over a long tract of sequence or over its whole sequence, without having densely biased regions such as the example above. CB regions can be part of well-studied classes of protein sequence, such as intrinsic disorder, structural proteins in cells and tissues, and functional amyloids and prions [1–3]. They may also give us clues to protein regions of yet uncharacterized biophysical types [3].

Programs to annotate protein CBs include SEG [4], CAST [5] and an algorithm by the author called LPS [3, 6, 7]. SEG annotates low-complexity sequences by performing a scan using thresholds for sequence entropy and a fixed window length. It is used for masking low-complexity sequences as part of the BLAST sequence alignment package [8]. Such masking has often been necessary since low-complexity sequences can lead to false inferences of protein homology. This is because of their simplicity. Similar low-complexity sequence can arise in unrelated proteins as these proteins evolve over millions of years. Another program CAST annotates low-complexity sequence by sequence alignment to homopeptides of the twenty common amino acids [5]. Also, the LPS algorithm used binomial probability to check for sequence regions of low probability, and was later developed to annotate CBs that arise from multiple amino-acid residue types [3, 6, 7]. The LPS algorithm has been applied successfully to the analysis of prions and prion-like proteins [1, 2, 9].

Here I introduce the program fLPS for the fast discovery of protein compositional biases. It builds on the LPS algorithm, but uses a number of new measures to substantially increase efficiency, chiefly through delaying or avoiding the actual calculation of probabilities unless/until it is absolutely necessary. It also has new functionality for varying user-defined parameters. It is quicker than other available programs for analysing CB, and is able to detect very mild biases over long stretches of sequence as well as pronounced biases over short stretches. The boundaries of CB regions are defined specifically from analysing the amounts of each individual amino-acid type in turn. fLPS outputs lists of CB regions labelled according to their amino-acid composition.

## Implementation

The program fLPS (pronounced ‘flips’) is written in standard C. The source code is distributed in the package. Also, there are two accessory scripts written in AWK. The program fLPS annotates single-residue, multiple-residue and whole-sequence compositional biases (CBs).

In the distribution, there are executables compiled for MacOSX (32-bit and 64-bit) and for Linux (64-bit only).

The output of fLPS is determined by eight command-line options, which are explained below in Results and discussion. The input files must contain protein sequences in standard FASTA format. The program can handle a FASTA-format file of any size.

The fLPS package is available from the project pages http://biology.mcgill.ca/faculty/harrison/flps.html, or https://github.com/pmharrison/flps, and is archived in Zenodo at https://zenodo.org/record/891004, or is also in Additional file 1. Examples of input and output files can be downloaded from the website http://biology.mcgill.ca/faculty/harrison/flps.html or are in Additional file 2.

## Results and discussion

### Overview of the algorithm

**Fig. 1:**
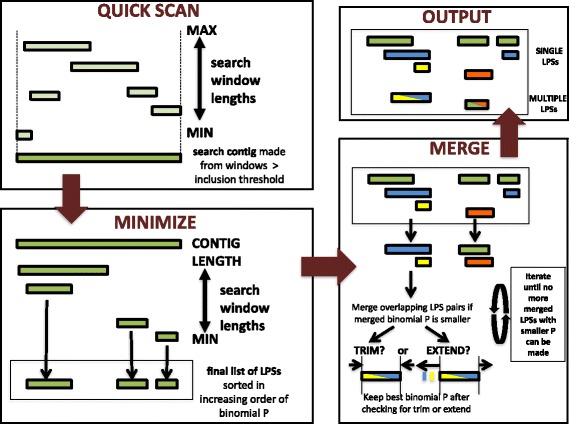
The algorithm. Three stages of bias annotation are depicted: *QUICK SCAN:* For each amino-acid residue type, from the maximum window size *M* down to the minimum *m*, the sequence is scanned for windows that have numbers of amino-acids greater than the expectation for a high binomial *P*-value threshold (=0.001). These windows are merged into a contig if they overlap each other. *MINIMIZE:* For each contig, the lowest-probability subsequences (LPSs) are computed by searching down from the contig length to the minimum *m*. *MERGE:* LPSs for different residue types are then sorted together in increasing order of binomial P-value and iteratively assessed for merger into multiple-residue LPSs. LPSs are merged if the merged LPS would have a lower P-value. This assessment entails checking whether the multiple-residue LPSs can be trimmed or extended, as depicted. Mergers of LPSs are assessed until no more can be performed. *OUTPUT:* Both single- and multiple-residue LPSs are output in increasing order of binomial P-value

The algorithm works through a process of probability minimization. First, sequences are quickly scanned for windows that are biased according to a high bias probability threshold (*P* = 0.001, but higher values could be used with some simple adjustments to the code) (Fig. 1). A range of window sizes are searched, down from the maximum *M* to minimum *m*, which can be user-defined. Windows are stored if they are biased enough and then if they overlap they are merged into a contig, i.e., a longer continuous sequence stretch. During the search process, for efficiency, a stored window is replaced with a smaller window if they have the same number of bias residues in them. At the end of this stage, there may be more than one contig for each residue type.

Second, each contig is searched exhaustively for low probability subsequences (LPSs), over a range of window sizes down from the length of the contig to the minimum *m* (Fig. 1). During the search process, to increase efficiency, all subsequences of length *L* are compared to all previously stored subsequences of length *L + 1*, and any such *L + 1* subsequences are de-selected according to simple rules about the fraction of biased residues. A final list of single-residue LPSs is produced by calculating the binomial *P*-values of the subsequences, sorting on increasing order of P-value, and progressively de-selecting overlappers that have higher P-values.

Third, LPSs for different residue types are iteratively assessed for possible merger (Fig. 1). After combining the lists of single-residue LPSs and sorting them on increasing order of P-value, pairs of LPSs with probabilities *P*_*1*_ and *P*_*2*_ are iteratively tested for merger, and kept as a multiple-residue LPS if the merged P-value *P*_*merge*_ < *P*_*1*_ and < *P*_*2*_. During the merger process, adjustments of the boundaries of the potential LPS to check for smaller values for *P*_*merge*_ are explored through trimming and extension. Trimming involves progressively receding from either or both endpoints of the potential multiple-residue LPS to search for a smaller *P*_*merge*_, until the minimum length *m* is reached. A similar search is performed using extension of the endpoints, except this search stops at either end when *P*_*merge*_ increases above its initial value (Fig. 1).

Finally, the program outputs all single-residue and multiple-residue LPSs, along with the results of a simple calculation of compositional biases over the whole protein sequence (Fig. 1).

### Parameters and output

**Fig. 2:**
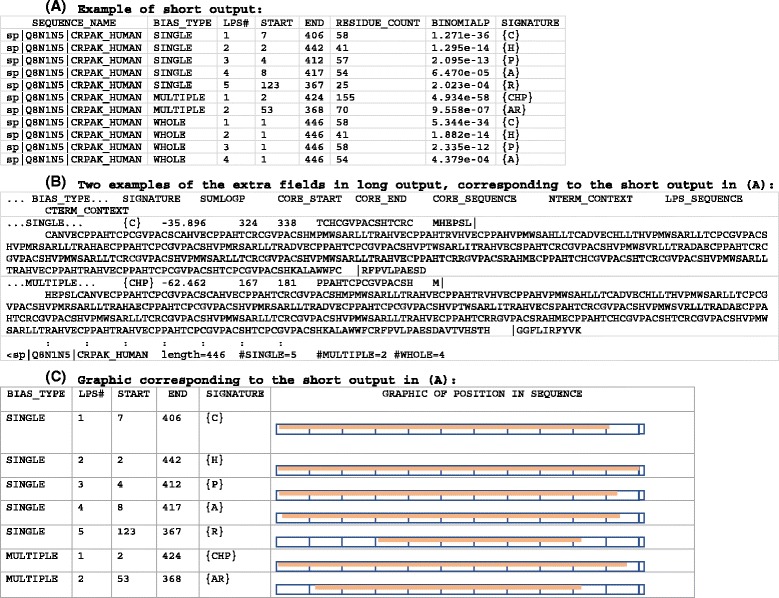
Output example. An example of the fLPS output in (**a**) short and (**b**) long formats, with a graphic of the LPSs in (**c**) (this is not part of the actual output of the program). The output is for protein CRPAK_HUMAN, human cysteine-rich PAK1 inhibitor. **a** The short format is: sequence name; type of bias (SINGLE-residue, MULTIPLE-residue or WHOLE-sequence); ordinal number of the LPS for the sequence (they are sorted in increasing order of binomial P-value); start residue in sequence; end residue in sequence; total number of bias residues in the LPS; binomial P-value for the LPS; CB signature (the single-letter amino-acid code of the residues is listed in order of precedence within curly brackets). **b** Two examples of the extra fields in long output, corresponding to the short output in (**a**). The long format has the additional fields: sum of log(P) (the sum of the log *P*-values of each of the constituent biases in the LPS, prior to merging); start residue of a core subsequence with the highest density of bias residues; end residue of the core subsequence; the core subsequence; up to 10 residues of N-terminal sequence context for the LPS; the LPS subsequence; up to 10 residues of C-terminal sequence context. Each LPS is listed on one line, except that in long format there is an optional summary footer that can be output using the ‘–d’ option. This begins with the ‘<’ symbol and contains these fields: sequence name; sequence length; number of SINGLE-residue LPSs; number of MULTIPLE-residue LPSs; number of WHOLE-sequence biases. For the long format in (**b**), for brevity most of the duplicated fields are omitted from the short format shown in (**a**). **c** A graphic of the LPSs. Bias type information, etc. as in (**a**)

There is depicted in Fig. 2a and b an example in both the short- and long-format fLPS outputs. In Fig. 2c, a graphic of each LPS is provided for perspective. Each LPS defines a CB region. Each has a CB signature, which is a list in curly brackets of the residue types contributing to the bias in order of their precedence. In the long format, a core sequence is displayed; this is simply the window of size minimum *m* that has the highest density of bias residues (if there is more than one with the highest density value, the window nearest the centre of the LPS is picked). These output formats are specified using the –o command-line option, with ‘–o short’ or ‘–o long’. A third output option is ‘–o masked’. This reproduces the input FASTA file, but with bias residues in LPSs masked with ‘Xs’.

There are eight other command-line options in fLPS. The –v option is for verbose runtime information, while –h displays a comprehensive help message. The –d option displays optional header and footer information in the output files. Option –s displays single-residue biases only. The user can define *m* and *M* the minimum and maximum window sizes with the –m and –M options, and a *P*-value threshold for the output with the –t option. This threshold is only used on output, not in the actual calculations. The final option (−c) specifies the background composition. Background ‘expected’ frequencies are necessary for the binomial P-value calculations. The user can specify ‘–c equal’ to assume equal expected frequencies of amino acids (=0.05). The default value ‘–c domains’ is for expected frequencies from a non-redundant set of protein domains taken from ASTRALSCOP (sequence identity threshold 40%) [10]. These frequencies thus give us low expectations for residues that are rare in structured protein domains (such as tryptophan and methionine), and high expectations for those that are abundant (such as alanine and serine). Users can also specify a background composition of their own making (‘–c filename’). A sensible approach is, if the input database is sufficiently large (i.e., thousands of proteins or more), to use the amino-acid composition of the database itself as the background composition. This can be calculated using a simple accessory script that is provided in the package. Using a proteome’s own composition ensures that some milder biased regions (with binomial *P*-values near to the threshold P-value) will be detected that might otherwise go undetected if another setting is used (e.g., such as ‘equal’ background frequencies for all of the amino-acids). However, for some analyses of compositional biases across multiple diverse data sets, it may be more appropriate to use the ‘equal’ background frequencies setting.

### Performance

**Fig. 3:**
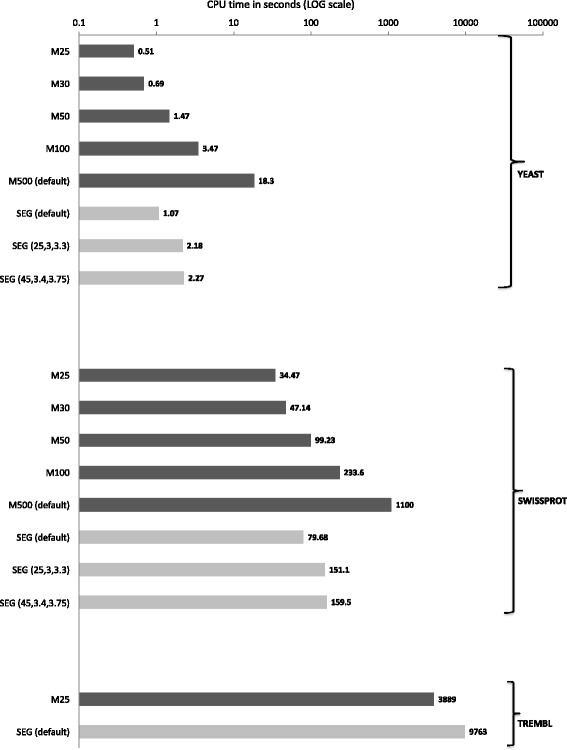
Timing: All three of the protein data sets analyzed here were downloaded from the UniProt website in August 2016 [11]. The programs were all run on an Intel Xeon CPU e5–1650 v2 @3.5GHz in an Apple Mac Pro that has 32GB of RAM and has installed in it MacOSX version 10.12.6. The program fLPS was tested for different maximum window size (*M*) values, and all other parameters set to defaults (dark grey bars). The CPU time in seconds is the sum of the user time and system time. For tests on the yeast (*Saccharomyces cerevisiae*) proteome (5782 sequences) and SwissProt (551,705 sequences) the time depicted is the mean from ten runs. For TrEMBL (65,378,749 sequences), the time for just one run is shown. Timings for SEG (light grey bars, default parameter values, and two other recommended parameter sets except for the TrEMBL run) are provided for comparison [4]

**Fig. 4:**
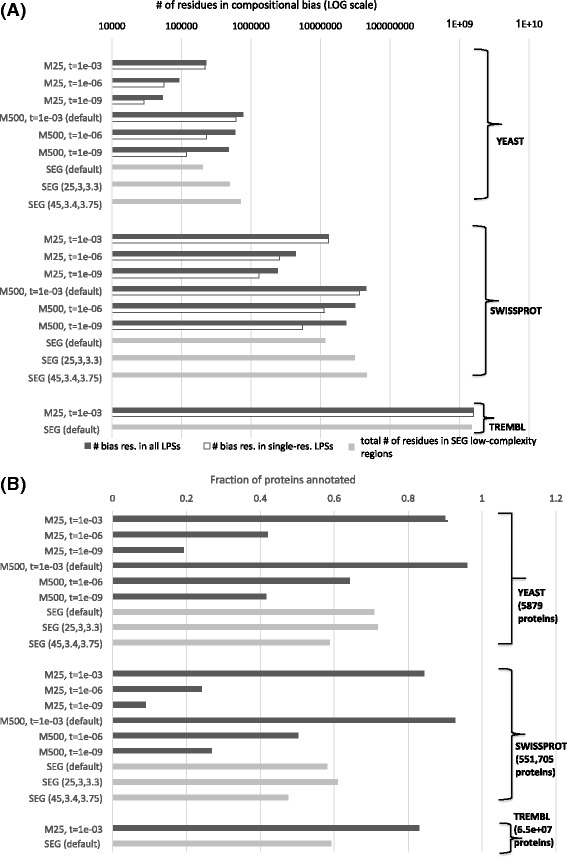
Amount of annotated bias. **a** Here, fLPS was run with the parameters listed, with all other parameters set to defaults. The total number of residues in multiple-residue LPSs (dark grey bars) and in single-residue regions (white bars) are shown. The total number of residues annotated as low-complexity by SEG (light grey, with default parameters, and up to two other recommended parameter sets) is provided for approximate comparison. **b** The fractions of the proteins from the databases that are annotated with CB. Programs are run as for part (A) and for Fig. 3

fLPS can readily handle databases with millions of sequences, as can be seen from the timing analysis for the TrEMBL database [11] (Fig. 3). Indeed, for a small *M* value (=25), it is >2 times quicker than the widely used SEG algorithm for low-complexity annotation [4], while at the same time annotating similar amounts of biased residues (Fig. 4a), that for the default P-value threshold (=1e-03) are distributed across more proteins in the database (Fig. 4b). Other combinations of *M* and *t* parameter values give widely different amounts of CB. The CB amounts found by either algorithm are conceptually different, i.e., fLPS distinguishes between single- and multiple-residue biases, and importantly, for fLPS the residues making up a bias are dispersed discontinuously, whereas this is not the case for SEG. For example, a sequence tract such as ‘...LXMXFGXXEXFXXWERT...’ may be annotated as biased by fLPS (represented here with the bias residues as ‘Xs’), whereas the corresponding SEG annotated region might be continuous, such as ‘...LKMXXXXXXXXXXWERT…’. Thus, the comparisons of CB amounts in Fig. 4a are very approximate. However, substantial percentages (> ~50% for some parameter settings) of CB regions found by either algorithm correspond to each other, but many detected regions are unique to either algorithm or do not have a simple correspondence (Additional file 3).

For small databases, such as the proteome of the yeast *S. cerevisiae*, fLPS takes just a few seconds, or even <1 s for small *M* values. This means that users can comfortably test for consistent annotations for different parameter sets, if they so wish.

### Examples of running the program

Here are some examples of running fLPS:

(i) *./fLPS –vm 10 –M 1000 –c YEAST.composition YEAST.fasta … …*

Here, fLPS analyses the file YEAST.fasta (containing the yeast proteome), outputting verbose runtime information, with minimum window size *m* = 10, maximum window size *M* = 1000. Also specified is the file YEAST.composition for background amino-acid frequencies, which the user has previously calculated from YEAST.fasta using the accessory AWK script included in the package.

(ii) *./fLPS –dst 1e-6 –c equal –o long uniprot.fasta … …*

This run analyses ‘uniprot.fasta’ for single-residue CBs only. It outputs the long format (which includes the biased region subsequences), and headers and footers are included. The output *P*-value threshold is 1e-6, and equal (=0.05) background amino-acid frequencies are used.

(iii) *./fLPS –m5 –M25 –t0.00001 –omasked uniprot.fasta … …*

Here, ‘uniprot.fasta’ is masked for CB regions using minimum window length *m* = 5 and maximum window length *M* = 25, and P-value threshold =0.00001.

Further examples are listed in the README bundled with the package, and in the help statement obtained by running *‘./fLPS –h’*.

### Usage

The program fLPS discovers CBs of any type, both short low-complexity tracts and longer regions with a compositional skew that are not detectable with short-window scans. In general, it is best to use the default parameter values (*m* = 15, *M* = 500, *t* = 0.001), which have been chosen through extensive trial and error experimentation. The default *M* = 500 is large enough that longer CB regions can be reliably detected, without extending the computation time too drastically (Fig. 3). If the user is specifically interested in very short CB regions a lower *m* value of 5 or 10 may be desirable.

**Fig. 5:**
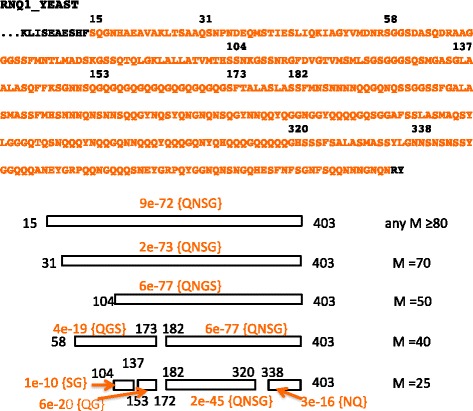
Behaviour of the algorithm with different *M* values. Here, as an example, I use the annotation of multiple-residue LPSs in the protein RNQ1_YEAST (Rnq1p, which underlies the [RNQ+]/[PIN+] prion in *S. cerevisiae* [19]). fLPS has been run with different maximum window size (*M*) values, and with other parameters set to defaults. With a sufficiently large *M* (≥80), one large LPS is annotated with signature {QNSG}. For smaller *M* values, this LPS is broken into smaller LPSs, as depicted by the boxes at the bottom of the figure. The endpoints of LPSs are numbered at the ends of a box. At the top of the figure, the LPS (for *M* ≥ 80) is highlighted in orange within the RNQ1_YEAST sequence. The endpoints of LPSs for different *M* values are labelled above the orange text, with the first numeral of the residue position aligned to the position in the sequence

In general, if lower *M* values are used, long CB regions might be broken up into shorter pieces, or in some cases may go undetected. This breaking up is illustrated for a CB region in the RNQ1 protein from the yeast *S. cerevisiae*, a protein that underlies the [RNQ+] prion (Fig. 5). With longer *M* values above a specific value (here *M* = 80), a longer CB region is annotated. Thus, to access such convergent boundary definitions and to find milder biases dispersed over long tracts, the default long *M* value =500 is appropriate. However, sometimes smaller CB domains (with the same or similar bias signatures, as in Fig. 5) may be evident from using a smaller *M* value. Such smaller tracts may be useful for construct design in experiments to delineate the functional parts of proteins and their CB domains.

Determining the boundaries of CB domains can be important for guiding experimental hypotheses. The examination of {NQ}-rich domains and their contiguous CB domains has been closely linked to experimentation on prion-forming domains in budding yeast for several years [12]. Prions in budding yeast are protein states that are propagated and inherited; most yeast prions are made of amyloid conformations that are passed onto further copies of the same proteins, and are usually formed from N/Q-rich domains [12]. Sup35p, which forms the [PSI+] prion [13], has a prion-forming domain which closely corresponds to a fLPS-identified {QYNG}-rich domain (residues 5 to 135, *P*-value = 1e-46). This is adjacent to an {EK}-rich CB region (residues 159 to 222, P-value = 9e-15), that corresponds to the ‘M-domain’ which can function in stabilizing [PSI+] prion fibers but is not necessary for prion formation/propagation [14]. For Swi1p, which forms the [SWI+] prion, although the prion-forming part was originally delimited to a span of ~300 residues that closely corresponds to an {N}-rich region detectable by fLPS (5–323, P-value = 6e-66), subsequently a very small subdomain of the CB region (residues 1–31) was found to be sufficient to propagate [SWI+] prions [15, 16]. Other Swi1p CB regions include an {AP}-rich region from residues 525 to 571 (P-value = 7e-09) within an intrinsically disordered stretch (as annotated by the default IUPRED program [17]). Conversely, for the prion-forming region of the transcriptional repressor Cyc8p, the exact boundaries have not been delimited within the region from residue 465 to 966 [18], but it splits into two contiguous CB regions, a {Q}-rich region delimited by fLPS spanning from 467 to 682 (P-value = 8e-76), and an {EST}-rich region (residues 699–952), both of which are predicted as intrinsically disordered (according to IUPRED [17]).

Different parameter values are appropriate if the user wishes to mask CB regions to examine homology in their absence. Masking may be desirable when searching for homologs of a protein that has a CB domain. Smaller maximums *M* ≤ 25, smaller minimums *m* ≤ 15, and lower thresholds 1e-06 ≤ *t* ≤ 1e-04 are suitable for this. Examining homology in the absence of CB regions may be critical for accurate multiple sequence alignment and phylogenetic tree construction, for specific proteins of interest to a user.

The output of fLPS makes it facile to search for similar CB regions in a protein database. The CB signature can be used to pick out such biases for any user query protein. An accessory script is provided in the package for searching in this way the output from running fLPS on a database. This script uses the difference in the sumLog(P) score (see Fig. 2 for definition) between the query and the database CB regions as a basic indicator of the difference in bias level. The script also allows for some permutation in the CB signature. Thereafter, a user could readily check for any functional associations for any particular type of CB region of interest. S/he could further restrict any biased regions by length, binomial *P*-value, sumLog(P) score, position in protein sequence, etc.

**Fig. 6:**
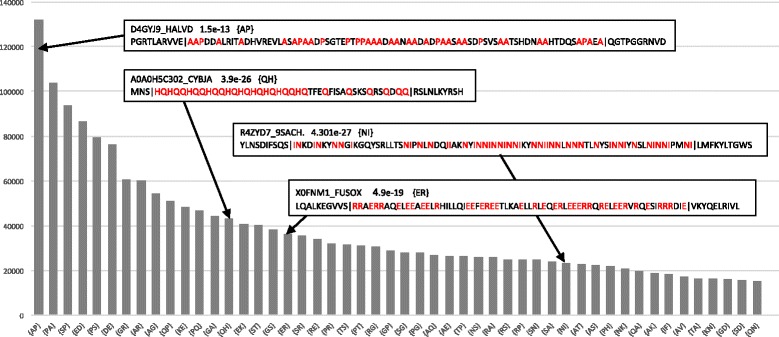
Most common short biased tracts in TrEMBL. The fifty most common CB regions of ≤100 residues in length and binomial *P*-value ≤1e-10, from the run of fLPS on TrEMBL with *M* = 25 and all other parameters at default values. The sequence names, binomial *P*-values and signatures of four random examples are shown, along with the LPSs delimited by ‘|’ with up to ten residues of sequence context at either end. TrEMBL was downloaded from the UniProt website in August 2016 [11]

Basic trends in large databases can also readily be analysed. As an example, we have performed a quick census for the TrEMBL database [11] of short, highly-biased CB tracts (≤100 residues long and binomial *P* ≤ 1e-10) (Fig. 6). The most common of these are {AP} and {PA} (an example is shown in the figure), followed closely by {SP}/{PS} and {ED}/{DE}. The alanine/proline co-occurrence may be linked to alanine codons (C-C-N, with N indicating any base) and proline codons (G-C-N) being very similar, likewise for the other common pairings.

### Summary of advances in the fLPS algorithm

fLPS comprises a number of advances on the LPS algorithm that was previously published [3, 6, 7]. fLPS and the previous LPS algorithm are both designed to annotate compositionally-biased regions in proteins, as defined above. Most significantly, the fLPS algorithm is substantially quicker (>80 times) when analyzing both multiple- and single-residue biases (using same processor for timings), and >20 times faster when analyzing single-residue ones only. This is because: (i) new measures have been introduced to delay probability calculations (as detailed above); (ii) analysis of multiple-residue biases has been quickened >1000-fold by switching to a trimming/extending method (as detailed above); (iii) the fLPS algorithm is in one executable that acts on database files of any size, whereas the previous algorithm analyzed only single sequences, and comprised two separate executables. Also, increased parameter ranges and choices are available in fLPS for window sizes, thresholds, and user-defined background frequencies. fLPS has three new different output formats, including output of databases masked for compositional biases.

## Conclusions

fLPS is an efficient tool for annotating CB regions. It annotates both short highly-biased tracts, and also longer regions that have a compositional skew. It can comfortably handle large databases, such as might arise from metagenomics projects. It can be applied to searching for proteins with similar CB regions, and for making functional inferences about CB regions for a protein of interest.

## Availability and requirements

**Project name** *:* fLPS.

**Project home page** *:* http://biology.mcgill.ca/faculty/harrison/flps.html and https://github.com/pmharrison/flps

**Archived version** *:* https://zenodo.org/record/891004

**Operating system** *:* executables compiled for MacOSX and Linux; source code is available to compile for other operating systems.

**Programming language** *:* C.

**Other requirements** *:* There are two accessory scripts written in AWK.

**License** *:* 3-clause BSD license.

**Restrictions to use by non-academics** *:* None.

## Additional files

Additional file 1: TAR archive file of the fLPS package. (GZ 322 kb)

Additional file 2: TAR archive file of example input and output files for fLPS. (GZ 10224 kb)

Additional file 3: Comparison of annotations from the fLPS and SEG programs. (DOCX 101 kb)

## Abbreviations

CB: Compositional bias or compositionally-biased
LPS: Low-probability subsequence
