# Supplementary material for: fLPS: Fast discovery of compositional biases for the protein universe
Source: BMC Bioinformatics. 2017 Nov 13;18:476. doi: 10.1186/s12859-017-1906-3 (PMC5684748; doi:10.1186/s12859-017-1906-3)
Supplement: Supplementary file 3 — Comparison of annotations from the fLPS and SEG programs. (DOCX 101 kb) [file 12859_2017_1906_MOESM3_ESM.docx]

**Additional File 2: Comparison of annotations from the fLPS and SEG programs.**

Comparing two algorithms that work using different principles is difficult. Chiefly, individual compositionally biased (CB) regions annotated by the fLPS program may correspond to several smaller CB regions found by SEG, or *vice versa*. Also, the determined boundaries can be quite different. Nevertheless, one would expect some convergence between the two algorithms. To check for this, the program fLPS was run on the yeast proteome with various parameters settings (minimum window *m*, maximum window *M* and probability threshold *t*). The resulting CB regions were then compared to those annotated by the SEG program (with default settings). The results are in Table S1 below. For each fLPS run the number of annotated CB regions is listed along with a tabulation of CB region overlaps with the SEG annotations. ‘Corresponding annotations’ are CB region annotations from one program whose two endpoints are near to the two endpoints of a CB region annotation from the other program. Specifically, the nearness of the endpoints is determined according to two margin values, ≤10 and ≤20 amino acid residues. Clearly, there is substantial overlap in the annotations, with ~40-60% of the annotations for either algorithm corresponding in this way for some fLPS parameter sets.

**Table S1: Comparison of fLPS and SEG annotations**

| **Settings for fLPS run:**  **–m15 –M25 –t1e-04** | | |
| --- | --- | --- |
| **Annotation totals 🡪** | **Total # of fLPS annotations = 15354** | **Total # of SEG annotations = 12851** |
| **Margin for endpoints** | **# of fLPS corresponding annotations** | **# of SEG corresponding annotations** |
| 10 | 4920 (32.0%) | 4281 (33.3%) |
| 20 | 7643 (49.8%) | 6358 (49.5%) |
| **Settings for fLPS run:**  **–m15 –M25 –t1e-05** | | |
| **Annotation totals 🡪** | **Total # of fLPS annotations =** 9138 | **Total # of SEG annotations =**  12851 |
| **Margin for endpoints** | **# of fLPS corresponding annotations** | **# of SEG corresponding annotations** |
| 10 | 3115 (34.1%) | 2791 (21.7%) |
| 20 | 4989 (54.6%) | 4421 (34.4%) |
| **Settings for fLPS run:**  **–m15 –M25 –t1e-06** | | |
| **Annotation totals 🡪** | **Total # of fLPS annotations =** 6021 | **Total # of SEG annotations =**  12851 |
| **Margin for endpoints** | **# of fLPS corresponding annotations** | **# of SEG corresponding annotations** |
| 10 | 2100 (34.9%) | 1896 (14.8%) |
| 20 | 3334 (55.4%) | 3053 (23.8%) |
| **Settings for fLPS run:**  **–m5 –M25 –t1e-04** | | |
| **Annotation totals 🡪** | **Total # of fLPS annotations =** 20823 | **Total # of SEG annotations =**  12851 |
| **Margin for endpoints** | **# of fLPS corresponding annotations** | **# of SEG corresponding annotations** |
| 10 | 6790 (32.6%) | 5900 (45.9%) |
| 20 | 9457 (45.4%) | 7711 (60.0%) |
| **Settings for fLPS run:**  **–m5 –M25 –t1e-05** | | |
| **Annotation totals 🡪** | **Total # of fLPS annotations =** 10902 | **Total # of SEG annotations =**  12851 |
| **Margin for endpoints** | **# of fLPS corresponding annotations** | **# of SEG corresponding annotations** |
| 10 | 4268 | 3847 |
| 20 | 6053 (54.6%) | 5334 (41.5%) |
| **Settings for fLPS run:**  **–m5 –M25 –t1e-06** | | |
| **Annotation totals 🡪** | **Total # of fLPS annotations =** 6743 | **Total # of SEG annotations =**  12851 |
| **Margin for endpoints** | **# of fLPS corresponding annotations** | **# of SEG corresponding annotations** |
| 10 | 2697 | 2452 |
| 20 | 3902 (57.9%) | 3558 (27.7%) |
